# Transmitted and pretreatment drug resistance among 69 HIV-1 CRFs in China: the first systematic analysis

**DOI:** 10.1080/22221751.2026.2673651

**Published:** 2026-06-09

**Authors:** Xiaoyi Yin, Bin Zhao, Minghui An, Danni Wang, Zhihan Liu, Hong Shang, Xiaoxu Han

**Affiliations:** a State Key Laboratory for Diagnosis and Treatment of Infectious Diseases, NHC Key Laboratory of AIDS Prevention and Treatment, National Clinical Research Center for Medical Auxiliary Technology (Laboratory Medicine), The First Hospital of China Medical University, China Medical University, Shenyang, People’s Republic of China; b Key Laboratory of AIDS Immunology, Chinese Academy of Medical Sciences, Shenyang, People’s Republic of China; c National Clinical Research Center for Medical Auxiliary Technology (Laboratory Medicine), Department of Laboratory Medicine, The First Hospital of China Medical University, China Medical University, Shenyang, People’s Republic of China

**Keywords:** Circulating recombinant forms (CRFs), pretreatment drug resistance (PDR), Transmitted drug resistance (TDR), v179d/E, China

## Abstract

In recent years, an increasing number of HIV-1 circulating recombinant forms (CRFs) have been identified in China. This study presented the first systematic analysis of the molecular epidemiological characteristics and drug resistance mutations (DRMs) profiles of all 69 CRFs identified in China up to 2025, comprising 13 first-generation, 45 second-generation, 10 third-generation, and one fourth-generation CRF . Several CRFs exhibited moderate transmitted drug resistance (5-15%) and elevated pretreatment drug resistance (10.8-55.1%). Notably, V179D/E were prevalent across multiple CRFs and, in combination with other DRMs, may contribute to reduced susceptibility to non-nucleoside reverse transcriptase inhibitors (NNRTIs). These findings provided critical surveillance data on DRMs and baseline data for NNRTIs selection in China, underscoring the necessity of phenotypic validation in novel CRFs harbouring natural DRMs.

Phylogenetic analyses have classified HIV-1 Group M into ten distinct subtypes (A–D, F–H, J–K), 158 circulating recombinant forms (CRFs) and numerous unique recombinant forms (URFs) [1]. Globally, CRFs and URFs accounted for 19.3% of HIV infections between 2016 and 2021 [2]. In China, CRFs constituted 88.9% of all HIV-1 infections, with CRF07_BC (39.1%), CRF01_AE (32.1%), CRF08_BC (9.2%), and CRF55_01B (2.4%) being the four most prevalent CRFs [3]. Notably, the proportions of CRF07_BC, CRF08_BC, CRF55_01B, other CRFs, and URFs increased significantly from 2004–2007 to 2020–2022 [4]. Furthermore, CRF07_BC has diversified into two sub-subtypes (07_N and 07_O), while CRF01_AE into eight sub-subtypes (01_C1-C8) in China [4, 5].

Advances in sequencing technologies have facilitated the identification of numerous novel CRFs derived from prevalent subtypes or sub-subtypes. Meanwhile, the prevalence of transmitted drug resistance (TDR) in China rose markedly from 2.6% in 2004–2007 to 7.8% in 2020–2022 [4], reaching 11.4% in 2023 [6]. Naturally occurring drug resistance mutations (DRMs) have been reported in CRF65_cpx and CRF55_01B identified and circulating in China [7]. However, no studies have yet conducted a systematic statistical analysis of the CRFs identified in China and their association with DRM characteristics.

This study compiled sequences and epidemiological data from antiretroviral therapy (ART)-naive individuals infected with the CRFs in China. Comprehensive data collection was finalized in May 2025 by retrieving HIV sequences from the Los Alamos HIV Sequence Database (https://www.hiv.lanl.gov/; data updated through October 14, 2024) and the China HIV Gene Sequence Data Platform (https://nmdc.cn/hiv/), and by conducting a systematic literature search through May 2025 to capture the latest research findings. Neighbor-joining trees were constructed using MEGA v7.0.14 to verify the components of CRF01_AE and CRF07_BC sub-subtypes [5] within the CRFs. Stanford University HIV Drug Resistance Database [8] was used to analyse DRMs. TDR was determined based on the WHO 2009 List of DRMs for Surveillance of Transmitted Drug Resistance [9]. The pretreatment drug resistance (PDR) rate was calculated by including only those DRMs or DRM combinations conferring at least low-level resistance.

As of 2025, a total of 69 CRFs have been first identified and named in China. Since 2000, when CRF07_BC and CRF08_BC were first reported, the number of CRFs detected in China has grown substantially. CRF55_01B was identified as the third CRF in 2013. Subsequently, nine CRFs were documented between 2013 and 2015, followed by 20 from 2016 to 2020, and another 38 from 2021 to 2025 (Figure 1(A)). Among these, CRFs involving CRF01_AE (*n* = 44) accounted for the largest proportion, followed by those involving CRF07_BC (*n* = 32), B (*n* = 31), C (*n* = 22), CRF55_01B (*n* = 9), and CRF08_BC (*n* = 4).

**Figure 1.**
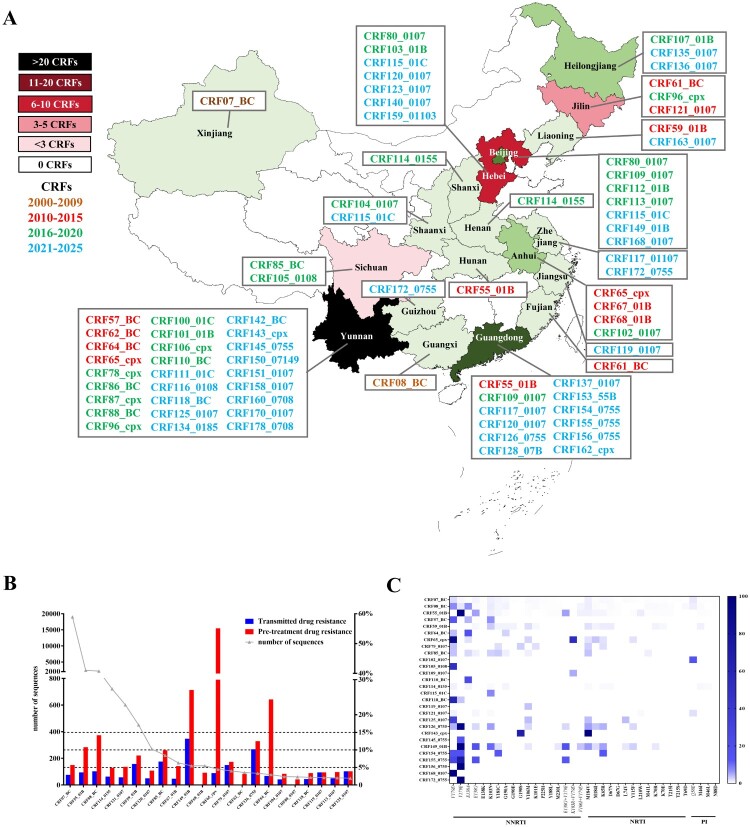
Spatiotemporal distribution and drug resistance of the HIV-1 circulated recombinant forms (CRFs) identified in China. (A) Geographic locations of the first identification of novel HIV-1 CRFs. Note that these locations represent the sites of original discovery/report rather than the current epidemiological prevalence or total burden of CRFs. (B) Distribution of transmitted drug resistance (TDR) and pretreatment drug resistance (PDR) among CRFs (only CRFs with >50 sequences were shown); and (C) percentage of various CRFs with PDR among drug resistance mutations (In bold: TDR mutations; only mutations with >10 sequences were shown). NNRTI, non-nucleoside reverse transcriptase inhibitors; NRTI, nucleoside reverse transcriptase inhibitors; PI, protease inhibitors.

Of all CRFs, 13 were identified as first-generation CRFs, 45 as second-generation CRFs, 10 as third-generation CRFs, and one (CRF150_07149) as a fourth-generation CRF . All first-generation CRFs were derived from subtype B from Thailand (B.TH) and C from India and were identified in people who inject drugs (PWID, *n* = 9) and heterosexuals (HET, *n* = 9). The majority of second-generation CRFs were recombinants involving CRF01_AE (*n* = 42, including 16 01_C5 and 11 01_C4), CRF07_BC (*n* = 26, including 21 07_N), or B (*n* = 16, including 7 B.TH), including initial CRFs based on CRF01_AE and B, followed by those involving CRF01_AE and CRF07_BC, as well as more complex CRF . Most third-generation CRFs were derived from CRF55_01B (*n* = 9) or CRF07_BC (*n* = 6, including 4 07_N). CRF150_07149 was the only fourth-generation CRF, based on recombination between 07_N and CRF149_01B. Given that the primary components of CRF, specifically 07_N (*n* = 26), 01_C5 (*n* = 17), 01_C4 (*n* = 12), and CRF55_01B (*n* = 9), were predominantly prevalent among men who have sex with men (MSM) [4], it followed that CRFs composed of these subtypes and sub-subtypes accounted for 90% (36/40) of all CRFs identified in MSM, the high-risk population with the largest number of CRF in China (Supplementary Table 1).

Yunnan (*n* = 28) and Guangdong (*n* = 12) have been identified as “hotspots” for CRFs in China, based on the regions where the CRFs were initially detected (Figure 1(A)). Situated along the drug trafficking route from the “Golden Triangle ,” Yunnan was not only the site of China's first HIV-1 epidemic in 1989 [10] but also continued to bear the heaviest HIV burden in China [11]. The long-term HIV prevalence has driven the emergence of multiple CRFs. Over time, the characteristics of CRFs detected in Yunnan have shifted in both genetic composition and affected populations. Prior to 2020, the identified CRFs (*n* = 13) were first- and second-generation recombinants mainly derived from subtypes B (92.3%) and C (92.3%), primarily detected among HET (76.9%) and PWID (69.2%). After 2020, the identified CRFs (*n* = 15) involved various generations, with backbones mainly from CRF01_AE (53.3%, 26.7% 01_C4) and CRF07_BC (53.3%, 40.0% 07_N) and were found mostly in HET (66.7%) and MSM (33.3%).

Guangdong, recognized as the province with the largest population and the most advanced economy in China, exhibited the highest number of migrants and was ranked among the top five provinces in HIV burden [11]. These factors likely contributed to the emergence and spread of novel CRFs. The majority of CRFs identified in Guangdong were detected after 2020 (91.7%). These included second-generation CRFs (*n* = 7), primarily based on CRF07_BC (85.7%, 71.4% 07_N) and CRF01_AE (85.7%, 57.1% 01_C5), and third-generation CRFs (*n* = 5), mainly based on CRF55_01B (100.0%) and CRF07_BC (80.0%, 40.0% 07_N). These strains were detected predominantly among MSM (83%) and HET (75%). Notably, 90% of third-generation CRFs (*n* = 10) were derived from CRF55_01B, initially identified among MSM in Guangdong in 2013 [12]. However, only 50% of third-generation CRFs were identified in Guangdong, suggesting that CRF55_01B has disseminated extensively across various regions of China and has undergone further local recombination. Consequently, regular molecular epidemiological surveillance of novel HIV-1 CRFs in Yunnan and Guangdong provinces was warranted.

Several CRFs now exhibited relatively high levels of TDR and PDR. Specifically, a moderate level of TDR (5.0-15.0%, *n* > 50 sequences) [13] was observed in CRF149_01B (13.2%, 19/144), CRF126_0755 (10.2%, 9/88), CRF85_BC (6.7%, 15/223), CRF59_01B (6.0%, 27/451), and CRF79_0107 (5.7%, 6/106). These observations indicated that DRMs accumulated under prolonged ART pressure have not only begun circulating among ART-naïve individuals but may also be inherited and further disseminated within these CRFs, underscoring the potential for sustained transmission and the need for broader awareness and continued surveillance. The high prevalence of PDR in CRF65_cpx (55.1%, 65/118), CRF149_01B (27.1%, 39/144), CRF64_BC (24.4%, 19/78), CRF08_BC (14.2%, 310/2179), CRF126_0755 (12.5%, 11/88), and CRF55_01B (10.8%, 262/2417) was attributed to the nature of the presence of numerous DRMs, such as V179D/E, particularly in combination with other mutations. These DRMs potentially confer varying degrees of resistance to non-nucleoside reverse transcriptase inhibitors (NNRTIs) (Figure 1(B), Supplementary Table 2).

Among 33 common DRMs identified in >10 sequences across 23 loci in 28 CRFs, V179E (10.4%, 2771/26547) and V179D (2.9%, 763/26547) were the most prevalent. V179E was a non-polymorphic mutation associated with weak selection pressure under efavirenz (EFV) [8]. A high prevalence of V179E was confirmed in CRF55_01B (98.6%), consistent with a previous report attributing its frequent occurrence (99.6%) to the founder effect [7]. Similarly high frequencies were observed in CRF126_0755 (95.5%, 84/88), CRF149_01B (99.3%, 143/144), CRF155_0755 (73.7%, 2382/2417), CRF156_0755 (100.0%, 5/5), and CRF172_0755 (100.0%, 4/4). This pattern likely reflected their origin from third-generation recombination events involving CRF55_01B. In contrast, V179D was a polymorphic accessory mutation occurring in approximately 1% of ART-naïve persons, and was occasionally selected under EFV pressure [8]. Previous studies have shown its widespread circulation in China [14, 15], providing a potential explanation for its high prevalence in the novel CRFs observed in this study, including CRF65_cpx (89.8%, 106/118), CRF105_0108 (50.0%, 3/6), CRF118_BC (60.3%, 35/58), CRF125_0107 (33.3%, 17/51), CRF155_0755 (26.3%, 5/19), and CRF168_0107 (100.0%, 4/4).

When presented in combination with other NNRTI DRMs, V179D/E can contribute to low-level reduced susceptibility to NNRTIs [8]. The detection of K103R + V179D combinations in CRF65_cpx (52.5%, 62/118) and E138G + V179E in CRF55_01B (5.6%, 136/2417) and CRF149_01B (16.0%, 23/144) (Figure 1(C)), which are known to confer moderate to high-level resistance to some NNRTIs, underscored the need for timely identification of these CRFs and baseline DRMs testing. Moreover, V179D/E may interfere with early virological response to NNRTI-based regimens (particularly those containing EFV) and increase the risk of acquired drug resistance [15]. However, phenotypic drug resistance data for these CRFs circulating in China remained limited. Consequently, it was imperative to conduct phenotypic testing of these CRFs to accurately assess drug susceptibility in the context of multiple polymorphic mutations (e.g. V179E/D). Such efforts would help guide optimal ART selection for infected individuals and ultimately improve ART outcomes.

This study had several limitations. First, subtype assignment based only on partial pol gene sequences may underestimate CRFs. Future studies incorporating recombination events from other genomic regions will therefore provide a more granular understanding of CRF evolution in China. Second, due to the limited number of sequences available for some CRFs (<100 sequences), the naturally polymorphic DRMs observed in this study required further validation with larger sample sizes.

In summary, this study systematically summarized the molecular epidemiology and DRM profiles of nearly all HIV-1 CRFs identified in China through 2025, identified critical surveillance DRMs, and provided an essential baseline of PDR to guide ART selection. Our results also revealed that certain naturally occurring DRMs, selected under prolonged ART pressure, can be inherited and further evolve within derived novel CRFs, warranting sustained monitoring. To translate these insights into clinical strategies and safeguard ART efficacy in China and globally, future efforts must improve sample representativeness and strengthen continuous, high-resolution surveillance.

## Supplementary Material

supplementary table 1.xlsx

supplementary table 2.xlsx

## Data Availability

The sequences presented in this study can be found in online repositories (Supplementary Table 1).
